# Geophysical water flows with constant vorticity and centripetal terms

**DOI:** 10.1007/s10231-020-00985-4

**Published:** 2020-05-02

**Authors:** Calin Iulian Martin

**Affiliations:** Faculty of Mathematics, University of Vienna, Oskar-Morgenstern-Platz 1, 1090 Vienna, Austria

**Keywords:** Coriolis terms, Centripetal terms, Vorticity, Three-dimensional water flows, 35Q31, 35Q35, 76B15, 76U05

## Abstract

We consider here three-dimensional water flows governed by the geophysical water wave equations exhibiting full Coriolis and centripetal terms. More precisely, assuming a constant vorticity vector, we derive a family of explicit solutions, in Eulerian coordinates, to the above-mentioned equations and their boundary conditions. These solutions are the only ones under the assumption of constant vorticity. To be more specific, we show that the components of the velocity field (with respect to the rotating coordinate system) vanish. We also determine a formula for the pressure function and we show that the surface, denoted $z=\eta (x,y,t)$, is time independent, but is not flat and can be explicitly determined. We conclude our analysis by converting to the fixed inertial frame, the solutions we obtained before in the rotating frame. It is established that, in the fixed frame, the velocity field is non-vanishing and the free surface is non-flat, being explicitly determined. Moreover, the system consisting of the velocity field, the pressure and the surface defining function represents explicit and exact solutions to the three-dimensional water waves equations and their boundary conditions.

## Introduction

We focus here on a problem of paramount importance in the study of geophysical fluid dynamics (GFD), namely, the determination of explicit analytical solutions to the full governing equations and their boundary conditions. The relative shortage of exact and/or explicit solutions is caused by the severe complications which are inherent in the (general) understanding of fluid flows. Indeed, thorough analytical investigations aiming at understanding GFD are quantitatively overwhelmed by experimental or observational studies which thrive on ad hoc modeling, numerical simulations or data driven approaches. Undoubtedly, the nonlinear character of the equations describing fluid flows, their three-dimensionality, the presence of whirls are features that, if taken simultaneously into account, greatly diminish the analytic tractability.

In addition to the already mentioned aspects, a study of GFD requires the inclusion of Coriolis effects, i.e., those arising from the Earth’s rotation, and which matter over sufficiently large time scales.

A series of accomplishments pertaining to water waves solutions in GFD has begun (relatively) recently with the analytical studies of Constantin [8–11] in which explicit solutions, portraying equatorially trapped waves propagating to the East, were presented. The flow pattern of these solutions is given in the Lagrangian framework by means of an essential extension [2, 9–11] to the three-dimensional case of the Gerstner wave solution [24] that bears relevance to the observed three-dimensional structure of geophysical flows. For other studies that use the Gerstner wave as a backbone to construct solutions to the GFD equations, we refer the reader to [18, 27, 32–34, 49–51]. The previous solutions refer to the *f*- or $\beta$-plane approximations and manifest a preferred propagation direction for the free surface and velocity field, being relevant to the description of the Equatorial Undercurrent (EUC) and of the Antarctic Circumpolar Current (ACC). Moreover, these solutions display the observed vertical structure, very much ignored in the reduced-gravity shallow water equations on the $\beta$-plane. Other exact solutions pertaining to EUC and ACC include also centripetal terms, cf. [14, 15, 26, 28–31, 42, 46–48], usually ignored in other studies.

While the Lagrangian description delivers important insights into the evolution of a particular particle, the Eulerian framework has the advantage that it provides the velocity field, the pressure and the free surface at any given time instant and any physical location. Following this realization, nonlinear studies of geophysical water flows have been performed recently by Constantin and Johnson [16, 19, 20]. A key role in the previous analyses is played by the vorticity, defined as the curl of the velocity field. The vorticity frames fundamental oceanic phenomena, which are inter-related (like upwelling/downwelling, zonal depth-dependent currents with flow reversal). The importance of the vorticity in the realistic modeling of ocean flows is highlighted in the very recent studies by Constantin and Johnson [19], Martin [44] and Wheeler [59]. On a related note, the vorticity serves the description of the vertical structure of the current profile in two-dimensional flows, cf. [3, 7, 23, 40, 53, 56].

It has become apparent that the vorticity also tremendously determines the dimensionality of the flow. Indeed, recent studies by Constantin [6], Constantin and Kartashova [4], Martin [41, 43] and Wahlen [58] show that gravity, capillary and capillary–gravity wave trains at the surface of water in a flow with constant non-zero vorticity with a flat bed can occur only if the flow is two dimensional and if the vorticity vector has only one non-vanishing component that points in the horizontal direction orthogonal to the direction of wave propagation. The same result remains true if the free surface is of most general type [43]. For related results concerning solitary waves, we refer the reader to the works by Craig [22] and Stuhlmeier [55]. In agreement with the conclusion of two-dimensionality of water flows with constant non-vanishing vorticity is the study by Xia and Francois [60] showing that in thick fluid layers, large-scale coherent structures can shear off the vertical eddies and reinforce the planarity of the flow.

In regard to the two-dimensionality, a somewhat similar result was achieved by Martin [45], where it was proved that a water flow satisfying the water wave equations with full Coriolis terms (but without centripetal ones) has also a two-dimensional character, but of different structure. Indeed, while in [4, 6, 41, 43, 58] the flow is proved to have non-vanishing vertical velocity, one non-vanishing horizontal velocity and a non-trivial free surface, the solution in [45] exhibits constant non-vanishing (time dependent) horizontal velocities, vanishing vertical velocity and flat surface.

In this paper, in addition to the problem considered in [45], we include the centripetal terms in the governing equations, while keeping, as in [45], the vorticity vector constant. The outcome is somewhat surprising: the velocity field necessarily has to vanish, but the free surface, while time independent, is non-flat, and the pressure has non-vanishing horizontal gradient, a circumstance that is also in striking contrast to [45]. We would like to point out that, while the velocity field is vanishing in the rotating frame, it is non-trivial in the inertial frame. Summarizing, we have derived a family of exact and explicit non-trivial solutions to the governing equations of GFD exhibiting full Coriolis and centripetal terms, in Eulerian coordinates, under the assumption of a constant vorticity vector. Moreover, the solutions we obtain are the only ones exhibiting constant vorticity. Writing the family of solutions we obtained in terms of the fixed inertial frame, we find that it has non-vanishing velocity field, the pressure and the free surface are also explicitly determined, the latter being non-flat. Furthermore, the vorticity vector is non-vanishing, which represents a marked difference, if compared with other studies of three-dimensional water waves in the Eulerian setting. Indeed, the irrotationality (that is, the vanishing of the vorticity vector) appears to have been an indispensable assumption for existence proofs [21, 25, 35, 36, 54] of three-dimensional water waves.

Although the solutions we present in the fixed frame display a certain simplicity, by way of having no time dependence, they are explicit and exact solutions to the full nonlinear governing Euler equations and their boundary conditions. Such solutions open up new perspectives for future nonlinear studies of rotational water flows by asymptotic or perturbative methods [17, 39].

While exact and explicit solutions depicting three-dimensional geophysical flows with vorticity were already obtained within the Lagrangian setting [10, 11, 18], the passage from the letter scenario to the Eulerian framework is quite a delicate matter. For nonlinear studies of rotational geophysical water flows, we refer the reader to the works of Constantin and Johnson [16, 19, 20] or Constantin and Monismith [18]. On a related note, recent rotational solutions (of piecewise constant vorticity) describing two-dimensional geophysical flows in Eulerian coordinates were obtained by Constantin and Ivanov [12, 13], and Ivanov [37]; see also the study by Basu [1].

## The Euler equations with full Coriolis and centripetal terms

We introduce here the governing equations for geophysical water waves. We choose a rotating framework $(\mathbf {i},\mathbf {j},\mathbf {k})$ with the origin on the Earth’s surface, at a point which, with respect to the fixed inertial frame, has the coordinates $(\cos \phi \cos \theta , \cos \phi \sin \theta , \sin \phi )$, where $\phi \in [-\frac{\pi }{2},\frac{\pi }{2}]$ denotes the angle of latitude and $\theta \in [0,2\pi ]$ represents the angle of longitude. Moreover, the *x*-axis is pointing horizontally due east, the *y*-axis horizontally due north and the *z*-axis upward. Since, the *x*-variable refers to longitude, the *y*-variable to the latitude and the *z*-variable denotes the local vertical, it is appropriate to assume a finite extent for *x*, *y*, *z*.

The governing equations for inviscid, homogeneous geophysical ocean flows are (up to the centripetal terms), (see e.g., [10, 52, 57]), the Euler’s equations

2.1 $$\begin{aligned} u_{t}+u u_{x}+v u_{y}+w u_{z} +2\omega w\cos \phi -2\omega v\sin \phi&=  -P_x,\nonumber \\ v_{t}+u v_{x}+v v_{y}+w v_{z} +2\omega u\sin \phi&=   -P_y,\nonumber \\ w_{t}+u w_{x}+v w_{y}+ w w_{z} -2\omega u\cos \phi&=   -P_z-g, \end{aligned}$$

and the equation of mass conservation

2.2 $$\begin{aligned} u_x+v_y+w_z=0, \end{aligned}$$

that are satisfied within the water flow domain bounded below by a rigid bed $z=-d (d>0)$ and above by the free surface $z=\eta (x,y,t)$. Here, *t* represents the time variable, $\phi$ is the latitude, $\omega = 7.29\cdot 10^{-5}$ rad $s^{-1}$ is the (constant) rotational speed of the Earth round the polar axis toward the east and *g* is the gravitational constant. The velocity field $u\mathbf {i}+v\mathbf {j}+w\mathbf {k}$, the pressure *P* and the free surface $\eta$ are assumed to be smooth enough. To the left-hand side of (2.1), we will add the centripetal terms

$$\begin{aligned} \vec {\omega }\times (\vec {\omega }\times \vec {r}) \end{aligned}$$

where $\vec {\omega }=\omega (\cos \phi )\vec {j}+\omega (\sin \phi )\vec {k}$ and $\vec {r}=x\vec {i}+y\vec {j}+(R+z)\vec {k}$, with $R\approx 6.3\cdot 10^3$ km being the Earth’s radius. More explicitly, we have

2.3 $$\begin{aligned} \vec {\omega }\times (\vec {\omega }\times \vec {r})&=\omega ^2 \left( -x\vec {i}+\left( -y\sin ^2\phi +(R+z)\sin \phi \cos \phi \right) \vec {j}\right. \nonumber \\&\quad \left. +\left( -(R+z)\cos ^2\phi +y\sin \phi \cos \phi \right) \vec {k}\right) . \end{aligned}$$

The specification of the water wave problem is completed by the boundary conditions pertaining to the free surface $z=\eta (x,y,t)$ and to the bed $z=-d$. These are the kinematic boundary conditions

2.4 $$\begin{aligned} w=\eta _t +u\eta _x+v\eta _y \quad \mathrm{on}\quad z=\eta (x,y,t) \end{aligned}$$

and

2.5 $$\begin{aligned} w=0\quad \mathrm{on}\quad z=-d, \end{aligned}$$

together with the dynamic boundary condition

2.6 $$\begin{aligned} P=P_\mathrm{atm}\quad \mathrm{on}\quad z=\eta (x,y,t), \end{aligned}$$

where $P_\mathrm{atm}$ denotes the constant atmospheric pressure. Condition (2.6) decouples the motion of the water from the motion of the air above it, cf. Constantin [5].

By a solution of the water wave problem (2.1)–(2.6), we understand a tuple $(u,v,w,P,\eta )$ whose components have sufficient regularity and satisfy (2.1)–(2.6).

We will use the vorticity vector field

2.7 $$\begin{aligned} \Omega =(w_{y}-v_{z},u_{z}-w_{x},v_{x}-u_{y})=:(\Omega _1,\Omega _2,\Omega _3) \end{aligned}$$

to catch the local flow rotation. According to the discussion in Constantin [8], the magnitude of the Equatorial Undercurrent’s relative vorticity (about $25\cdot 10^{-3}$ m $s^{-1}$) is much larger than that of the planetary vorticity $2\omega \sim 1.46\cdot 10^{-4} s^{-1}$. Therefore, throughout the paper, we will make the assumptions

2.8 $$\begin{aligned} \Omega _2+2\omega \cos \phi \ne 0\quad \mathrm{and}\quad \Omega _3+2\omega \sin \phi \ne 0. \end{aligned}$$

We state now the first result which shows that the velocity field is time independent under the assumption of constant vorticity vector $\Omega$.

### **Theorem 2.1**

*We assume that* $\phi \ne 0$ *and that the vorticity vector* $\Omega$ *is constant throughout the flow and also satisfies* (2.8). *Then, the velocity field* $u\vec {i}+v\vec {j}+w\vec {k}$ *has the property that* *u* *and* *v* *depend only on the time* *t*, *while* $w=0$ *throughout the water flow*.

### *Proof*

We start by passing to the curl of equations in (2.1) having the centripetal terms (2.3) adjoined. However, since the centripetal term $\vec {\omega }\times (\vec {\omega }\times \vec {r})$ is curl-free and using also that $\Omega _1,\Omega _2,\Omega _3$ **are constants**, we obtain

2.9 $$\begin{aligned}&\Omega _1 u_x+(\Omega _2+2\omega \cos \phi )u_y+(\Omega _3+2\omega \sin \phi )u_z=0,\nonumber \\&\Omega _1 v_x+(\Omega _2+2\omega \cos \phi )v_y+(\Omega _3+2\omega \sin \phi )v_z=0,\nonumber \\&\Omega _1 w_x+(\Omega _2+2\omega \cos \phi )w_y+(\Omega _3+2\omega \sin \phi )w_z=0. \end{aligned}$$

Equations (2.9) along with a suitable modification of a subtle argument initiated by Constantin [6] will allow us to considerably simplify the flow structure. We proceed by noticing that we have from the last equation in (2.9) (along with assumption (2.8) that *w* is constant in a direction that is not parallel to the horizontal plane $z=-d$. By (2.5), we obtain that *w* vanishes throughout the flow. The definition of the vorticity vector delivers the equations

2.10 $$\begin{aligned} u_z(x,y,z,t)&=   \Omega _2\quad \mathrm{for}\,\,\mathrm{all}\quad x,y,z,t, \end{aligned}$$

2.11 $$\begin{aligned} v_z(x,y,z,t)&=   -\Omega _1\quad \mathrm{for}\,\,\mathrm{all}\quad x,y,z,t, \end{aligned}$$

from which we infer that there are functions $(x,y,t)\rightarrow \widetilde{u}(x,y,t)$ and $(x,y,t)\rightarrow \widetilde{v}(x,y,t)$ such that

2.12 $$\begin{aligned} u(x,y,z,t)&=\widetilde{u}(x,y,t)+\Omega _2 z,\nonumber \\ v(x,y,z,t)&=\widetilde{v}(x,y,t)-\Omega _1 z, \end{aligned}$$

for all *x*, *y*, *z*, *t* for which $-d\le z\le \eta (x,y,t)$. Moreover, the functions $\widetilde{u}$ and $\widetilde{v}$ satisfy the equation

$$\begin{aligned} \widetilde{u}_x(x,y,t)+\widetilde{v}_y(x,y,t)=0, \,\,\mathrm{for}\,\,\mathrm{all}\,\, x,y,t, \end{aligned}$$

which yields the existence of a function $(x,y,t)\rightarrow \psi (x,y,t)$ such that

2.13 $$\begin{aligned} \widetilde{u}(x,y,t)=\psi _y(x,y,t)\quad \mathrm{and} \quad \widetilde{v}(x,y,t)=-\psi _x(x,y,t), \end{aligned}$$

for all *x*, *y*, *t*.

Returning now to the system (2.9), we see that its first two equations are rewritten as

2.14 $$\begin{aligned}&\Omega _1 \psi _{xy}+(\Omega _2+2\omega \cos \phi )\psi _{yy} +(\Omega _3+2\omega \sin \phi )\Omega _2=0,\nonumber \\&\Omega _1 \psi _{xx}+(\Omega _2+2\omega \cos \phi )\psi _{xy} +(\Omega _3+2\omega \sin \phi )\Omega _1=0. \end{aligned}$$

Moreover, from the third component of the vorticity vector (2.7), we obtain

2.15 $$\begin{aligned} \Delta \psi =-\Omega _3. \end{aligned}$$

Solving the system of equations consisting of (2.14) and (2.15), we obtain

2.16 $$\begin{aligned} \psi _{yy}&=\frac{2\omega \Omega _1^2\sin \phi -\widetilde{\Omega _2} \widetilde{\Omega _3}\Omega _2}{\Omega _1^2+\widetilde{\Omega _2}^2}=:A \nonumber \\ \psi _{xy}&=-\frac{\Omega _1(\Omega _2\widetilde{\Omega _3}+2\omega \widetilde{\Omega _2}\sin \phi )}{\Omega _1^2+\widetilde{\Omega _2}^2}=:B\nonumber \\ \psi _{xx}&=\frac{2\omega \widetilde{\Omega _2}(\Omega _2\sin \phi -\Omega _3\cos \phi ) -\Omega _1^2\Omega _3-2\omega \Omega _1^2\sin \phi }{\Omega _1^2+\widetilde{\Omega _2}^2}=:C \end{aligned}$$

where

$$\begin{aligned} \widetilde{\Omega _2}:=\Omega _2+2\omega \cos \phi , \quad \widetilde{\Omega _3}:=\Omega _3+2\omega \sin \phi . \end{aligned}$$

Therefore, by means of (2.16), there exist functions $t\rightarrow a(t),t\rightarrow b(t), t\rightarrow k(t)$ such that

2.17 $$\begin{aligned} \psi (x,y,t)=\frac{A}{2}y^2+Bxy+\frac{C}{2}x^2+a(t)y+b(t)x +k(t)\,\,\mathrm{for}\,\,\mathrm{all}\,\,x,y,t. \end{aligned}$$

By means of the previous formula and employing also (2.12), we find that

2.18 $$\begin{aligned} \widetilde{u}(x,y,t)&=   Ay+Bx+a(t), \end{aligned}$$

2.19 $$\begin{aligned} \widetilde{v}(x,y,t)&=   -Cx+By-b(t). \end{aligned}$$

Invoking now the boundedness of $\widetilde{u}$ and $\widetilde{v}$ and utilizing (2.18) and (2.19), we infer that

$$\begin{aligned} A=B=C=0. \end{aligned}$$

We claim now that $\Omega _1=0$. To prove this claim, we assume for the sake of contradiction that $\Omega _1\ne 0$. Then, since $B=0$, we conclude from the formula for *B* in (2.16) that

$$\begin{aligned} \Omega _2\widetilde{\Omega _3}+2\omega \widetilde{\Omega _2}\sin \phi =0. \end{aligned}$$

Multiplying the previous equation with $\widetilde{\Omega _2}$ and adding the result to the equation $A=0$, we obtain the relation

$$\begin{aligned} 2\omega (\Omega _1^2+\widetilde{\Omega _2}^2)\sin \phi =0, \end{aligned}$$

which is impossible, since we assumed $\Omega _1\ne 0$ and we work under the assumption that $\phi \ne 0$. Therefore, the assumption $\Omega _1\ne 0$ cannot be true, hence, the claim that $\Omega _1=0$ is proved. From $A=0$, we see now that $\Omega _2\widetilde{\Omega _2}\widetilde{\Omega _3}=0$. Since $\widetilde{\Omega _2}\widetilde{\Omega _3}\ne 0$, we conclude now that $\Omega _2=0$. Recalling that $C=0$, we obtain from the third formula in (2.16) that $\Omega _3\cos \phi =0$, from which we derive that $\Omega _3=0$. Equations (2.10)–(2.11) now imply that

2.20 $$\begin{aligned} u_z(x,y,z,t)=v_z(x,y,z,t)=0\,\,\mathrm{for}\,\,\mathrm{all}\,\,x,y,z,t. \end{aligned}$$

With the latter equation and employing also the previous findings concerning the vanishing of $\Omega _1,\Omega _2,\Omega _3$, we see that equation (2.9) reduces to

$$\begin{aligned} 2\omega u_y\cos \phi =2\omega v_y\cos \phi =0, \end{aligned}$$

which obviously implies that

2.21 $$\begin{aligned} u_y(x,y,z,t)=v_y(x,y,z,t)=0\,\,\mathrm{for}\,\,\mathrm{all}\,\,x,y,z,t. \end{aligned}$$

Availing now of $\Omega _3=0$ and of the incompressibility condition (2.2) (both used in conjunction with (2.21)), we find that

2.22 $$\begin{aligned} u_x(x,y,z,t)=v_x(x,y,z,t)=0\,\,\mathrm{for}\,\,\mathrm{all}\,\,x,y,z,t. \end{aligned}$$

We notice now that (2.20)–(2.22) imply that *u* and *v* are functions of *t* only. $\square$

Our goal now will be to find more details about *u*(*t*) and *v*(*t*) and to provide formulas for the free surface $\eta$ and for the pressure *P*.

### **Theorem 2.2**

*It holds that* $u=v\equiv 0$,

$$\begin{aligned} \eta _t(x,y,t)=0\,\,\mathrm{for}\,\,\mathrm{all}\,\, x,y,t, \end{aligned}$$

*and the pressure* *P* *is given as*

2.23 $$\begin{aligned} P(x,y,z,t)&=\frac{\omega ^2}{2}x^2+\frac{\omega ^2\sin ^2\phi }{2}y^2 -(\omega ^2\sin \phi \cos \phi ) y(R+z) -g(R+z)\nonumber \\&\quad +\frac{\omega ^2\cos ^2\phi }{2}(R+z)^2+c, \end{aligned}$$

*for all* *x*, *y*, *t* *and* $-d\le z\le \eta (x,y)$, *where* *c* *is an arbitrary constant and* $\eta (x,y)$ *satisfies a second degree algebraic equation*.

### *Proof*

Using now all the inferences pertaining to the velocity field, found above, we observe that the Euler’s equations become

2.24 $$\begin{aligned}&u^{\prime }(t)-2\omega v(t)\sin \phi -\omega ^2 x=-P_x(x,y,z,t)\nonumber \\&\quad v^{\prime }(t)+2\omega u(t)\sin \phi -\omega ^2(\sin \phi )^2 y +\omega ^2(\sin \phi \cos \phi )(R+z)=-P_y(x,y,z,t)\nonumber \\&\quad -2\omega u(t)\cos \phi -\omega ^2(\cos \phi )^2 (R+z) +\omega ^2(\sin \phi \cos \phi )y=-P_z(x,y,z,t)-g \end{aligned}$$

equality which is true for all (*x*, *y*, *t*) and *z* such that $-d\le z\le \eta (x,y,t)$.

Integrating the above equations yields

2.25 $$\begin{aligned} P(x,y,z,t)&=-\left( u^{\prime }(t)-2\omega v(t)\sin \phi \right) x +\frac{\omega ^2}{2}x^2\nonumber \\&\quad -\left( v^{\prime }(t)+2\omega u(t)\sin \phi \right) y +\frac{\omega ^2(\sin \phi )^2}{2}y^2\nonumber \\&\quad -\left( \omega ^2\sin \phi \cos \phi \right) y(R+z) +\left( 2\omega u(t)\cos \phi -g\right) (R+z)\nonumber \\&\quad +\frac{\omega ^2(\cos \phi )^2}{2}(R+z)^2+c(t). \end{aligned}$$

In the sequel, we will exploit the kinematic boundary condition (2.4) and the dynamic boundary condition (2.6). Note that, differentiating the dynamic boundary condition with respect to *t*, *x*, *y*, respectively, we obtain that for all *x*, *y*, and *t*, it holds that

2.26 $$\begin{aligned}&P_t(x,y,\eta (x,y,t),t)+P_z(x,y,\eta (x,y,t),t)\eta _t(x,y,t)=0\nonumber \\&P_x(x,y,\eta (x,y,t),t)+P_z(x,y,\eta (x,y,t),t)\eta _x(x,y,t)=0\nonumber \\&P_y(x,y,\eta (x,y,t),t)+P_z(x,y,\eta (x,y,t),t)\eta _y(x,y,t)=0 \end{aligned}$$

We claim now that

2.27 $$\begin{aligned} P_z(x,y,\eta (x,y,t),t)\ne 0\,\,\mathrm{for}\,\,\mathrm{all}\,\,x,y,t. \end{aligned}$$

To prove the claim, we assume that there are $x_0,y_0$ and $t_0$ such that

2.28 $$\begin{aligned} P_z(x_0,y_0,\eta (x_0,y_0,t_0),t_0)=0. \end{aligned}$$

Differentiating the second equation in (2.26) with respect to *x*, we find that

2.29 $$\begin{aligned}&P_{xx}(x,y,\eta (x,y,t),t)+P_{xz}(x,y,\eta (x,y,t),t)\eta _x(x,y,t)\nonumber \\&\quad +\left( P_{zx}(x,y,\eta (x,y,t),t)+P_{zz}(x,y,\eta (x,y,t),t) \eta _x(x,y,t)\right) \eta _x(x,y,t)\nonumber \\&\quad +P_z(x,y,\eta (x,y,t),t)\eta _{xx}(x,y,t)=0. \end{aligned}$$

Using the assumption (2.28) and that $P_{xz}\equiv 0$, we have from the previous formula that

$$\begin{aligned} \omega ^2 +\omega ^2 (\cos ^2\phi ) \eta _x^2(x_0,y_0,t_0)=0, \end{aligned}$$

clearly impossible. This renders the claim (2.27) true. Hence, we can express now $\eta _t,\eta _x$ and $\eta _y$ from (2.26), and by means of the kinematic boundary condition (2.4) (taking also into account that $w=0$), we obtain that

2.30 $$\begin{aligned} P_t(x,y,\eta (x,y,t),t)+u(t)P_x(x,y,\eta (x,y,t),t)+v(t)P_y(x,y,\eta (x,y,t),t)=0 \end{aligned}$$

for all *x*, *y*, *t*. To explicitate the previous equation, we note that

2.31 $$\begin{aligned} P_t&=-\left( u^{\prime \prime }(t)-2\omega v^{\prime }(t)\sin \phi \right) x -\left( v^{\prime \prime }(t)+2\omega u^{\prime }(t)\sin \phi \right) y\nonumber \\&\quad +2\omega u^{\prime }(t)(\cos \phi )(R+z) +c^{\prime }(t) \end{aligned}$$

Therefore, using also the formulas for $P_x$ and $P_y$ , we have from (2.30) that for all *x*, *y*, *t* it holds

2.32 $$\begin{aligned}&{[}\omega ^2 u(t)-\left( u^{\prime \prime }(t)-2\omega v^{\prime }(t) \sin \phi \right) ]x+[\omega ^2 v(t) (\sin \phi )^2-\left( v^{\prime \prime }(t) +2\omega u^{\prime }(t)\sin \phi \right) ]y\nonumber \\&\quad +c^{\prime }(t)-u(t)u^{\prime }(t)-v(t)v^{\prime }(t) +\omega \cos \phi (2u^{\prime }(t)-\omega v(t)\sin \phi )[R+\eta (x,y,t)]=0. \end{aligned}$$

We assume now that there is a $t_0$ such that

2.33 $$\begin{aligned} 2u^{\prime }(t_0)-\omega v(t_0)\sin \phi \ne 0. \end{aligned}$$

Differentiating two times with respect to *x* in (2.32) we obtain

$$\begin{aligned} \omega \cos \phi (2u^{\prime }(t_0)-\omega v(t_0)\sin \phi ) \eta _{xx}(x,y,t_0)=0\,\,\mathrm{for}\,\,\mathrm{all}\,\,x,y. \end{aligned}$$

Clearly, our assumption (2.33) yields now that $\eta _{xx}(x,y,t_0)=0$ for all *x*, *y*. Differentiating now with respect to *x* in the second equation of (2.26), we have that

$$\begin{aligned} P_{xx}\Big |_{z=\eta (x,y,t_0)}+P_{zx}\Big |_{z=\eta (x,y,t_0)}\cdot \eta _x+ P_{zz}\Big |_{z=\eta (x,y,t_0)}\cdot \eta _x^2 +P_z\Big |_{z=\eta (x,y,t_0)}\cdot \eta _{xx}(x,y,t_0)=0, \end{aligned}$$

expression which, using that $P_{xz}\equiv 0$ and $\eta _{xx}(x,y,t_0)=0$, becomes

$$\begin{aligned} \omega ^2 +\omega ^2(\cos ^2\phi )\eta _x^2=0. \end{aligned}$$

The latter is clearly not possible. Therefore, the assumption that there is a $t_0$ such that $2u^{\prime }(t_0)-\omega v(t_0)\sin \phi \ne 0$ is false. Hence,

2.34 $$\begin{aligned} 2u^{\prime }(t)-\omega v(t)\sin \phi =0\,\,\mathrm{for}\,\,\mathrm{all}\,\,t, \end{aligned}$$

and we immediately see that (2.32) becomes

2.35 $$\begin{aligned}&[\omega ^2 u-\left( u^{\prime \prime }-2\omega v^{\prime }\sin \phi \right) ]x +[\omega ^2 v (\sin \phi )^2-\left( v^{\prime \prime } +2\omega u^{\prime }\sin \phi \right) ]y\nonumber \\&\quad +c'-uu^{\prime }-vv^{\prime }=0 \end{aligned}$$

for all *x*, *y*, *t*. From (2.35), we obtain that

2.36 $$\begin{aligned} \omega ^2 u(t)-\left( u^{\prime \prime }(t)-2\omega v^{\prime }(t) \sin \phi \right) =0\,\,\mathrm{for}\,\,\mathrm{all}\,\,t \end{aligned}$$

and

2.37 $$\begin{aligned} \omega ^2 v(t) (\sin \phi )^2-\left( v^{\prime \prime }(t)+2\omega u^{\prime }(t)\sin \phi \right) =0\,\,\mathrm{for}\,\,\mathrm{all}\,\,t \end{aligned}$$

Using (2.34), we wee that (2.37) becomes

$$\begin{aligned} v^{\prime \prime }\equiv 0, \end{aligned}$$

which implies that $v^{\prime }$ is a constant. Then, from (2.34), we have that $u^{\prime \prime }$ is a constant. Since $v^{\prime }$ and $u^{\prime \prime }$ are constants, we infer from (2.36) that *u* is a constant, that is $u^{\prime }\equiv 0$. From (2.34), we then get that $v\equiv 0$, and then from (2.36), we obtain that $u\equiv 0$. From the kinematic boundary condition (2.4), we see immediately that $\eta _t\equiv 0$.

One last consequence of (2.32) is that $c^{\prime }(t)=0$ for all *t*. That is, the function $t\rightarrow c(t)$ is a constant, which we denote with *c*.

We return now to the formula for the pressure (2.25). Since $u=v\equiv 0$, we have that for all *x*, *y*, *z*, *t* it holds

2.38 $$\begin{aligned} P(x,y,z,t)&=\frac{\omega ^2}{2}x^2+\frac{\omega ^2\sin ^2\phi }{2}y^2 -(\omega ^2\sin \phi \cos \phi ) y(R+z) -g(R+z)\nonumber \\&\quad +\frac{\omega ^2\cos ^2\phi }{2}(R+z)^2+c, \end{aligned}$$

where *c* is an arbitrary constant.

We will determine now $\eta$ from the dynamic boundary condition (2.6). Indeed, from (2.6), we have

2.39 $$\begin{aligned}&\frac{\omega ^2\cos ^2\phi }{2}(R+\eta (x,y))^2-[\omega ^2 (\sin \phi \cos \phi ) y+g](R+\eta (x,y))\nonumber \\&\quad +\frac{\omega ^2}{2}x^2+\frac{\omega ^2\sin ^2\phi }{2}y^2 +c-P_\mathrm{atm}=0, \end{aligned}$$

which we treat as a second degree algebraic equation in the unknown $R+\eta (x,y)$. The discriminant of the above equation is

2.40 $$\begin{aligned} \Delta =[\omega ^2 (\sin \phi \cos \phi ) y+g]^2-\omega ^2\cos ^2\phi \left[ \omega ^2 x^2+\omega ^2(\sin ^2\phi )y^2+2c-2P_\mathrm{atm}\right] . \end{aligned}$$

Owing to the finite extent of (*x*, *y*), we can choose a constant *c* such that $\omega ^2 x^2+\omega ^2(\sin ^2\phi )y^2\le 2P_\mathrm{atm}-2c$. For any such *c*, we have that $\Delta \ge 0$ and (2.39) has a unique positive solution. More precisely, since $R+\eta (x,y)>0$,

2.41 $$\begin{aligned} R+\eta (x,y)=\frac{\omega ^2 (\sin \phi \cos \phi ) y+g +\sqrt{\Delta }}{\omega ^2\cos ^2\phi }. \end{aligned}$$

$$\square $$

In the following, we will derive the absolute velocity in the fixed inertial frame (*I*, *J*, *K*), where $I=(0,0,1),\, J=(0,1,0),\, K=(0,0,1)$. Associated with this velocity profile, we will find a pressure function that together with a formula for the surface build a family of explicit and exact solutions to the full nonlinear Euler equations and their boundary conditions.

### **Proposition 2.3**

*While the velocity field* $u\vec {i}+v\vec {j}+w\vec {k}$ *vanishes in the rotating frame, it is non-vanishing in the fixed frame* (*I*, *J*, *K*). *More precisely, denoting with*

$$\begin{aligned} \tilde{U}(X,Y,Z,t),\,\,\tilde{V}(X,Y,Z,t),\,\,\tilde{W}(X,Y,Z,t) \end{aligned}$$

*the components of the velocity field in the inertial frame* (*I*, *J*, *K*), *we have that*

$$\begin{aligned} \tilde{U}(X,Y,Z,t)=-\omega Y, \,\tilde{V}(X,Y,Z,t) =\omega X,\,\tilde{W}(X,Y,Z,t)\equiv 0. \end{aligned}$$

*Furthermore, there is a unique (up to a constant) function* $(X,Y,Z)\rightarrow \tilde{P}(X,Y,Z)$ *and a unique (up to a constant) function* $(X,Y)\rightarrow \tilde{\eta }(X,Y)$ *representing the surface such that*

2.42 $$\begin{aligned}&\tilde{U}_t+\tilde{U}\tilde{U}_X+\tilde{V}\tilde{U}_Y +\tilde{W}\tilde{U}_Z=-\tilde{P}_ X,n\nonumber \\&\tilde{V}_t+\tilde{U}\tilde{V}_X+\tilde{V}\tilde{V}_Y +\tilde{W}\tilde{V}_Z=-\tilde{P}_ Y,\nonumber \\&\tilde{W}_t+\tilde{U}\tilde{W}_X+\tilde{V}\tilde{W}_Y +\tilde{W}\tilde{W}_Z=-\tilde{P}_Z-g, \end{aligned}$$

2.43 $$\begin{aligned}&\tilde{P}(X,Y,\eta (X,Y))=P_\mathrm{atm}, \end{aligned}$$

*and*

2.44 $$\begin{aligned} \tilde{W}(X,Y,\eta (X,Y))=\tilde{U}(X,Y,\eta (X,Y)) \tilde{\eta }_X(X,Y)+\tilde{V}(X,Y,\eta (X,Y))\tilde{\eta }_Y(X,Y). \end{aligned}$$

*Lastly, the gradient of the function* $(X,Y,Z)\rightarrow gZ+\tilde{P}(X,Y,Z)$ *coincides with the term* $P_x\vec {i}+P_y\vec {j}+(g+P_z)\vec {k}$, *with the latter written in the* (*I*, *J*, *K*) *system*.

### *Proof*

Let us denote with $\frac{dX}{dt}I+\frac{dY}{dt}J+\frac{dZ}{dt}K$ the absolute velocity in the inertial frame (*I*, *J*, *K*). We then have that

2.45 $$\begin{aligned} \frac{dX}{dt}I+\frac{dY}{dt}J+\frac{dZ}{dt}K=U\vec {i}+V\vec {j}+W\vec {k} \end{aligned}$$

where

2.46 $$\begin{aligned} \left( \begin{array}{c} U\\ V\\ W \end{array}\right)&=   \left( \begin{array}{c} u-\omega y\sin \phi +\omega z\cos \phi +\omega R\cos \phi \\ v+\omega x\sin \phi \\ w -\omega x\cos \phi \end{array}\right) \nonumber \\&=   \left( \begin{array}{c} -\omega y\sin \phi +\omega z\cos \phi +\omega R\cos \phi \\ +\omega x\sin \phi \\ -\omega x\cos \phi \end{array}\right) \end{aligned}$$

by the vanishing of *u*, *v*, *w*. Since

2.47 $$\begin{aligned}&x=-X\sin \alpha +Y\cos \alpha \nonumber \\&y=-X\sin \phi \cos \alpha -Y\sin \phi \sin \alpha +Z\cos \phi \nonumber \\&z=-R+X\cos \phi \cos \alpha +Y\cos \phi \sin \alpha +Z\sin \phi \end{aligned}$$

(where $\alpha =\theta +\omega t$) we obtain

2.48 $$\begin{aligned} \left( \begin{array}{c} U\\ V\\ W \end{array}\right) =\left( \begin{array}{c} \omega X\cos \alpha +\omega Y\sin \alpha \\ -\omega X\sin \alpha \sin \phi +\omega Y\cos \alpha \sin \phi \\ \omega X\sin \alpha \cos \phi -\omega Y\cos \alpha \cos \phi \end{array}\right) . \end{aligned}$$

We find now $\tilde{U},\tilde{V},\tilde{W}$ such that

2.49 $$\begin{aligned} U\vec {i}+V\vec {j}+W\vec {k}=\tilde{U}I+\tilde{V}J+\tilde{W}K. \end{aligned}$$

We use that

2.50 $$\begin{aligned}&\vec {i}=- I\sin \alpha +J\cos \alpha \nonumber \\&\vec {j}=-(I\cos \alpha +J\sin \alpha )\sin \phi +K\cos \phi \nonumber \\&\vec {k}=(I\cos \alpha +J\sin \alpha )\cos \phi +\sin \phi K \end{aligned}$$

and (2.48) to obtain that

2.51 $$\begin{aligned} \frac{\tilde{U}(X,Y,Z)}{\omega }&=-\sin \alpha (X\cos \alpha +Y\sin \alpha ) -(-X\sin \alpha \sin \phi +Y\cos \alpha \sin \phi )\sin \phi \cos \alpha \nonumber \\&\quad +(X\sin \alpha \cos \phi -Y\cos \alpha \cos \phi )\cos \phi \cos \alpha =-Y, \end{aligned}$$

that is

2.52 $$\begin{aligned} \tilde{U}(X,Y,Z)=-\omega Y. \end{aligned}$$

Similar calculations lead to

$$\begin{aligned} \tilde{V}(X,Y,Z)=\omega X,\quad \mathrm{and}\quad \tilde{W}(X,Y,Z)=0, \end{aligned}$$

for all *X*, *Y*, *Z*. Clearly, the velocity field in the inertial frame (*I*, *J*, *K*)

2.53 $$\begin{aligned} \tilde{U}(X,Y,Z),\tilde{V}(X,Y,Z),\tilde{W}(X,Y,Z))=(-\omega Y,\omega X,0) \end{aligned}$$

does not vanish, and moreover,

$$\begin{aligned} \tilde{U}_X+\tilde{V}_Y+\tilde{W}_Z=0 \end{aligned}$$

for all *X*, *Y*, *Z*.

Furthermore, it holds that

2.54 $$\begin{aligned} \tilde{U}_t+\tilde{U}\tilde{U}_X+\tilde{V}\tilde{U}_Y+\tilde{W}\tilde{U}_Z&=-\omega ^2 X,\nonumber \\ \tilde{V}_t+\tilde{U}\tilde{V}_X+\tilde{V}\tilde{V}_Y+\tilde{W}\tilde{V}_Z&=-\omega ^2 Y,\nonumber \\ \tilde{W}_t+\tilde{U}\tilde{W}_X+\tilde{V}\tilde{W}_Y+\tilde{W}\tilde{W}_Z&=0. \end{aligned}$$

Denoting with $\tilde{P}$ the pressure in the fixed frame (*I*, *J*, *K*), we have that

2.55 $$\begin{aligned} \tilde{P}_X=\omega ^2 X,\quad \tilde{P}_Y=\omega ^2 Y,\quad \tilde{P}_Z=-g, \end{aligned}$$

from which we infer that there is a function $t\rightarrow f(t)$ such that

2.56 $$\begin{aligned} \tilde{P}(X,Y,Z,t)=\frac{\omega ^2}{2}(X^2+Y^2)-gZ +f(t) \,\,\mathrm{for}\,\,\mathrm{all}\,\,X,Y,Z,t. \end{aligned}$$

We will prove in the following that $f^{\prime }(t)=0$ for all *t*. Let us denote $\tilde{\eta }(X,Y,Z,t)$ the expression of the free surface in the coordinates *X*, *Y*, *Z* in the fixed frame (*I*, *J*, *K*). In the dynamic boundary condition

2.57 $$\begin{aligned} \tilde{P}(X,Y,\tilde{\eta }(X,Y,t),t)=P_\mathrm{atm}\,\,\mathrm{for}\,\,\mathrm{all}\,\,X,Y,t, \end{aligned}$$,

we differentiate by *t*, *X* and *Y*, respectively, and obtain that for all *X*, *Y*, *t* it holds

2.58 $$\begin{aligned}&\tilde{P}_t(X,Y,\tilde{\eta }(X,Y,t),t)+\tilde{P}_Z(X,Y,\tilde{\eta } (X,Y,t),t)\tilde{\eta }_t(X,Y,t)=0,\nonumber \\&\tilde{P}_X(X,Y,\tilde{\eta }(X,Y,t),t)+\tilde{P}_Z(X,Y,\tilde{\eta } (X,Y,t),t)\tilde{\eta }_X(X,Y,t)=0,\nonumber \\&\tilde{P}_Y(X,Y,\tilde{\eta }(X,Y,t),t)+\tilde{P}_Z(X,Y,\tilde{\eta } (X,Y,t),t)\tilde{\eta }_Y(X,Y,t)=0. \end{aligned}$$

From (2.55) and (2.58), we have that

2.59 $$\begin{aligned} \tilde{\eta }_t(X,Y,t)&=\frac{\tilde{P}_t(X,Y,\tilde{\eta }(X,Y,t),t)}{g},\nonumber \\ \tilde{\eta }_X(X,Y,t)&=\frac{\tilde{P}_X(X,Y,\tilde{\eta }(X,Y,t),t)}{g} =\frac{\omega ^2 X}{g},\nonumber \\ \tilde{\eta }_Y(X,Y,t)&=\frac{\tilde{P}_Y(X,Y,\tilde{\eta }(X,Y,t),t)}{g} =\frac{\omega ^2 Y}{g}. \end{aligned}$$

We note now that, since $\tilde{W}=0$, the kinematic boundary condition on the free surface $Z=\tilde{\eta }(X,Y,t)$ asserts that for all *X*, *Y*, *t* the relation

2.60 $$\begin{aligned} 0=\tilde{\eta }_t (X,Y,t)+\tilde{U}(X,Y,\tilde{\eta }(X,Y,t),t)\tilde{\eta }_X +\tilde{V}(X,Y,\tilde{\eta }(X,Y,t),t)\tilde{\eta }_Y \end{aligned}$$

holds. The latter relation together with (2.53) and (2.59) yield that

2.61 $$\begin{aligned} \tilde{\eta }_t(X,Y,t)=0\,\,\mathrm{for}\,\,\mathrm{all}\,\,X,Y,t. \end{aligned}$$

Writing now the dynamic boundary condition (2.57), we have

2.62 $$\begin{aligned} \frac{\omega ^2}{2}(X^2+Y^2)-g\tilde{\eta }(X,Y) +f(t)=P_\mathrm{atm} \,\,\mathrm{for}\,\,\mathrm{all}\,\,X,Y,t. \end{aligned}$$

The output of the differentiation with respect to *t* in the above relation is that the function $t\rightarrow f(t)$ is, in fact, a constant, which we denote again with *f*. Therefore,

2.63 $$\begin{aligned} \tilde{P}(X,Y,Z,t)=\frac{\omega ^2}{2}(X^2+Y^2)-gZ +f\,\,\mathrm{for} \,\,\mathrm{all}\,\,X,Y,Z,t. \end{aligned}$$

Consequently, the surface defining function $\tilde{\eta }$ is given as

2.64 $$\begin{aligned} \tilde{\eta }(X,Y)=\frac{1}{g}\cdot \left( \frac{\omega ^2}{2}(X^2+Y^2) -P_\mathrm{atm}+f\right) \,\,\mathrm{for}\,\,\mathrm{all}\,\,X,Y. \end{aligned}$$

It is also easy to see that $\tilde{\eta }$, given by the above formula, satisfies the surface kinematic condition (2.60). A computation shows that the gradient of the function $(X,Y,Z)\rightarrow gZ+\tilde{P}(X,Y,Z)$ equals $P_x\vec {i}+P_y\vec {j}+(g+P_z)\vec {k}$.

Summarizing, we have proved that the system consisting of $\tilde{U},\tilde{V},\tilde{W},\tilde{P},\tilde{\eta }$ given in (2.53),(2.63) and (2.64) satisfy the Euler equations, the equation of mass conservation and the kinematic and dynamic boundary conditions.

### **Remark 2.4**

We would like to note that the vorticity vector associated with the previous flow equals $(0,0,\tilde{V}_X-\tilde{U}_Y)=(0,0,2\omega )$, which is non-vanishing, a feature that is in striking contrast with existence type results [21, 25, 35, 36, 54], where irrotationality seems to be of vital importance for proving existence of solutions describing three-dimensional water flows with free surface.

### **Remark 2.5**

We find it interesting that the flow solution $\tilde{U},\tilde{V},\tilde{W},\tilde{P},\tilde{\eta }$ presented in Proposition 2.3 also satisfies the Navier–Stokes system

2.65 $$\begin{aligned} \frac{D\tilde{U}}{Dt}&=-\frac{1}{\rho }\tilde{P}_X+\nu \Delta \tilde{U},\nonumber \\ \frac{D\tilde{V}}{Dt}&=-\frac{1}{\rho }\tilde{P}_Y+\nu \Delta \tilde{V},\nonumber \\ \frac{D\tilde{W}}{Dt}&=-\frac{1}{\rho }\tilde{P}_Z -g+\nu \Delta \tilde{W}, \end{aligned}$$

($\nu$ being the kinematic viscosity and $\rho$ the density), and also the normal stress condition [38]

2.66 $$\begin{aligned}&P-\frac{2\rho \nu \left( \tilde{\eta }_X^2\tilde{U}_X+\tilde{\eta }_Y^2\tilde{V}_Y -\tilde{\eta }_X(\tilde{U}_Z+\tilde{W}_X) -\tilde{\eta }_Y(\tilde{V}_Z+\tilde{W}_Y) +\tilde{\eta }_X\tilde{\eta }_Y(\tilde{U}_Y+\tilde{V}_X)+\tilde{W}_Z \right) }{1+\tilde{\eta }_X^2+\tilde{\eta }_Y^2}\nonumber \\&\quad =P_\mathrm{atm} \end{aligned}$$

as well as the tangential stress conditions [38], that in the absence of wind are written as

2.67 $$\begin{aligned} \tilde{\eta }_X(\tilde{V}_Z+\tilde{W}_Y)-\tilde{\eta }_Y(\tilde{U}_Z+\tilde{W}_X) +2\tilde{\eta }_X\tilde{\eta }_Y (\tilde{U}_X-\tilde{V}_Y)-(\tilde{\eta }_X^2 -\tilde{\eta }_Y^2)(\tilde{U}_Y+\tilde{V}_X)=0 \end{aligned}$$

and

2.68 $$\begin{aligned}&2\tilde{\eta }_X^2(\tilde{U}_X-\tilde{W}_Z)+2\tilde{\eta }_Y^2(\tilde{V}_Y -\tilde{W}_Z)+2\tilde{\eta }_X\tilde{\eta }_Y(\tilde{U}_Y+\tilde{V}_X)\nonumber \\&\quad +(\tilde{\eta }_X^2+\tilde{\eta }_Y^2-1)\left( \tilde{\eta }_X(\tilde{U}_Z +\tilde{W}_X) +\tilde{\eta }_Y(\tilde{V}_Z+\tilde{W}_Y)\right) =0. \end{aligned}$$

$$\square $$

## References

[1] Basu B (2019). On an exact solution of a nonlinear three-dimensional model in ocean flows with equatorial undercurrent and linear variation in density. Discrete Contin. Dyn. Syst. Ser. A.

[2] Constantin A (2001). On the deep water wave motion. J. Phys. A.

[3] Constantin A, Strauss W (2004). Exact steady periodic water waves with vorticity. Commun. Pure Appl. Math..

[4] Constantin A, Kartashova E (2009). Effect of non-zero constant vorticity on the nonlinear resonances of capillary water waves. Europhys. Lett..

[5] Constantin, A.: Nonlinear water waves with applications to wave-current interactions and tsunamis, volume 81. In: CBMS-NSF Regional Conference Series in Applied Mathematics. Society for Industrial and Applied Mathematics (SIAM), Philadelphia, PA (2011)

[6] Constantin A (2011). Two-dimensionality of gravity water flows of constant non-zero vorticity beneath a surface wave train. Eur. J. Mech. B/Fluids.

[7] Constantin A, Escher J (2011). Analyticity of periodic traveling free surface water waves with vorticity. Ann. Math..

[8] Constantin A (2012). On the modelling of equatorial waves. Geophys. Res. Lett..

[9] Constantin A (2012). An exact solution for equatorially trapped waves. J. Geophys. Res. Oceans.

[10] Constantin A (2013). Some three-dimensional nonlinear equatorial flows. J. Phys. Oceanogr..

[11] Constantin A (2014). Some nonlinear, equatorially trapped, nonhydrostatic internal geophysical waves. J. Phys. Oceanogr..

[12] Constantin A, Ivanov RI (2015). A hamiltonian approach to wave-current interactions in two-layer fluids. Phys. Fluids.

[13] Constantin A, Ivanov RI (2019). Equatorial wave–current interactions. Commun. Math. Phys..

[14] Constantin A, Johnson RS (2016). An exact, steady, purely azimuthal equatorial flow with a free surface. J. Phys. Oceanogr..

[15] Constantin A, Johnson RS (2016). An exact, steady, purely azimuthal flow as a model for the Antarctic Circumpolar Current. J. Phys. Oceanogr..

[16] Constantin A, Johnson RS (2017). A nonlinear, three-dimensional model for ocean flows, motivated by some observations of the Pacific Equatorial Undercurrent and thermocline. Phys. Fluids.

[17] Constantin A, Johnson RS (2017). Large gyres as a shallow-water asymptotic solution of Euler’s equation in spherical coordinates. Proc. R. Soc. A Math. Phys. Eng. Sci..

[18] Constantin A, Monismith S (2017). Gerstner waves in the presence of mean currents and rotation. J. Fluid Mech..

[19] Constantin A, Johnson RS (2018). Steady large-scale ocean flows in spherical coordinates. Oceanography.

[20] Constantin A, Johnson RS (2019). On the nonlinear, three-dimensional structure of equatorial oceanic flows. J. Phys. Oceanogr..

[21] Craig W, Nicholls DP (2000). Travelling two and three dimensional capillary gravity water waves. SIAM J. Math. Anal..

[22] Craig W (2002). Non-existence of solitary water waves in three dimensions. Recent developments in the mathematical theory of water waves (Oberwolfach, 2001). R. Soc. Lond. Philos. Trans. Ser. A Math. Phys. Eng. Sci..

[23] Escher J (2012). Regularity of rotational travelling water waves. Philos. Trans. R. Soc. A Math. Phys. Eng. Sci..

[24] Gerstner F (1809). Theorie der Wellen samt einer daraus abgeleiteten Theorie der Deichprofile. Ann. Phys..

[25] Groves MD, Haragus M (2003). A bifurcation theory for three-dimensional oblique travelling gravity–capillary water waves. J. Nonlinear Sci..

[26] Haziot S (2019). Study of an elliptic partial differential equation modelling the Antarctic Circumpolar Current. Discrete Contin. Dyn. Syst. Ser. A..

[27] Henry D (2013). An exact solution for equatorial geophysical water waves with an underlying current. Eur. J. Mech. B Fluids.

[28] Henry D (2016). Equatorially trapped nonlinear water waves in a $$\beta $$-plane approximation with centripetal forces. J. Fluid Mech..

[29] Henry D (2017). A modified equatorial $$\beta $$-plane approximation modelling nonlinear wave–current interactions. J. Differ. Equ..

[30] Henry D, Martin CI (2019). Free-surface, purely azimuthal equatorial flows in spherical coordinates with stratification. J. Differ. Equ..

[31] Henry D, Martin CI (2019). Azimuthal equatorial flows with variable density in spherical coordinate. Arch. Ration. Mech. Anal..

[32] Ionescu-Kruse D (2015). An exact solution for geophysical edge waves in the f-plane approximation. Nonlinear Anal. Real World Appl..

[33] Ionescu-Kruse D (2015). An exact solution for geophysical edge waves in the $$\beta $$-plane approximation. J. Math. Fluid Mech..

[34] Ionescu-Kruse D (2017). Exact steady Azimuthal edge waves in rotating fluids. J. Math. Fluid Mech..

[35] Iooss G, Plotnikov PI (2009). Small divisor problem in the theory of three-dimensional water gravity waves. Mem. Am. Math. Soc..

[36] Iooss G, Plotnikov PI (2011). Asymmetrical three-dimensional travelling gravity waves. Arch. Ration. Mech. Anal..

[37] Ivanov RI (2017). Hamiltonian model for coupled surface and internal waves in the presence of currents. Nonlinear Anal. Real World Appl..

[38] Johnson RS (1997). A Modern Introduction to the Mathematical Theory of Water Waves. Cambridge Texts in Applied Mathematics.

[39] Johnson RS (2018). Applications of the ideas and techniques of classical fluid mechanics to some problems in physical oceanography. Philos. Trans. R. Soc. A Math. Phys. Eng. Sci..

[40] Jonsson IG, Le Méhauté B (1990). Wave–current interactions. The Sea: Ocean Engineering Science.

[41] Martin CI (2017). Resonant interactions of capillary–gravity water waves. J. Math. Fluid Mech..

[42] Martin CI (2017). On the existence of free-surface azimuthal equatorial flows. Appl. Anal..

[43] Martin CI (2018). Non-existence of time-dependent three-dimensional gravity water flows with constant non-zero vorticity. Phys. Fluids.

[44] Martin CI (2018). On the vorticity of mesoscale ocean currents. Oceanography.

[45] Martin CI (2019). Constant vorticity water flows with full Coriolis term. Nonlinearity.

[46] Martin CI, Quirchmayr R (2019). Explicit and exact solutions concerning the Antarctic Circumpolar Current with variable density in spherical coordinates. J. Math. Phys..

[47] Martin CI, Quirchmayr R (2019). A steady stratified purely Azimuthal flow representing the Antarctic Circumpolar Current. Monatsh. Math..

[48] Marynets K (2019). On the modeling of the flow of the Antarctic Circumpolar Current. Monatsh. Math..

[49] Matioc A-V (2012). An exact solution for geophysical equatorial edge waves over a sloping beach. J. Phys..

[50] Matioc A-V (2013). Exact geophysical waves in stratified fluids. Appl. Anal..

[51] Matioc A-V (2014). On the particle motion in geophysical deep water waves traveling over uniform currents. Q. Appl. Math..

[52] Pedlosky J (1979). Geophysical Fluid Dynamics.

[53] Peregrine DH (1976). Interactions of water waves and currents. Adv. Appl. Mech..

[54] Reeder J, Shinbrot M (1981). Three-dimensional, nonlinear wave interaction in water of constant depth. Nonlinear Anal..

[55] Stuhlmeier R (2012). On constant vorticity flows beneath two-dimensional surface solitary waves. J. Nonlinear Math. Phys..

[56] Thomas GP, Klopman G, Hunt JN (1997). Wave–current interactions in the nearshore region. Gravity Waves in Water of Finite Depth.

[57] Vallis GK (2006). Atmospheric and Oceanic Fluid Dynamics.

[58] Wahlén E (2014). Non-existence of three-dimensional travelling water waves with constant non-zero vorticity. J. Fluid Mech..

[59] Wheeler MH (2018). Simplified models for equatorial waves with vertical structure. Oceanography.

[60] Xia H, Francois N (2017). Two-dimensional turbulence in three-dimensional flows. Phys. Fluids.

